# High depth, whole-genome sequencing of cholera isolates from Haiti and the Dominican Republic

**DOI:** 10.1186/1471-2164-13-468

**Published:** 2012-09-11

**Authors:** Rachel Sealfon, Stephen Gire, Crystal Ellis, Stephen Calderwood, Firdausi Qadri, Lisa Hensley, Manolis Kellis, Edward T Ryan, Regina C LaRocque, Jason B Harris, Pardis C Sabeti

**Affiliations:** 1 Computer Science and Artificial Intelligence Laboratory (CSAIL), Massachusetts Institute of Technology (MIT), Cambridge, MA, USA; 2 Broad Institute of MIT and Harvard, Cambridge, MA, USA; 3 Center for Systems Biology, Department of Organismic and Evolutionary Biology, Harvard University, Cambridge, MA, USA; 4 Division of Infectious Diseases, Massachusetts General Hospital, Boston, MA, USA; 5 Department of Medicine, Harvard Medical School, Boston, MA, USA; 6 International Centre for Diarrheal Disease Research, Dhaka, Bangladesh; 7 Viral Therapeutics, United States Army Institute of Infectious Disease, Fort Detrick, MD, USA; 8 Department of Immunology and Infectious Diseases, Harvard School of Public Health, Cambridge, MA, USA; 9 Department of Pediatrics, Harvard Medical School, Boston, MA, USA

**Keywords:** Whole-genome sequencing, *Vibrio cholerae*, Haitian cholera epidemic, Microbial evolution

## Abstract

**Background:**

Whole-genome sequencing is an important tool for understanding microbial evolution and identifying the emergence of functionally important variants over the course of epidemics. In October 2010, a severe cholera epidemic began in Haiti, with additional cases identified in the neighboring Dominican Republic. We used whole-genome approaches to sequence four *Vibrio cholerae* isolates from Haiti and the Dominican Republic and three additional *V. cholerae* isolates to a high depth of coverage (>2000x); four of the seven isolates were previously sequenced.

**Results:**

Using these sequence data, we examined the effect of depth of coverage and sequencing platform on genome assembly and identification of sequence variants. We found that 50x coverage is sufficient to construct a whole-genome assembly and to accurately call most variants from 100 base pair paired-end sequencing reads. Phylogenetic analysis between the newly sequenced and thirty-three previously sequenced *V. cholerae* isolates indicates that the Haitian and Dominican Republic isolates are closest to strains from South Asia. The Haitian and Dominican Republic isolates form a tight cluster, with only four variants unique to individual isolates. These variants are located in the CTX region, the SXT region, and the core genome. Of the 126 mutations identified that separate the Haiti-Dominican Republic cluster from the *V. cholerae* reference strain (N16961)*,* 73 are non-synonymous changes, and a number of these changes cluster in specific genes and pathways.

**Conclusions:**

Sequence variant analyses of *V. cholerae* isolates, including multiple isolates from the Haitian outbreak, identify coverage-specific and technology-specific effects on variant detection, and provide insight into genomic change and functional evolution during an epidemic.

## Background

Following the 2010 earthquake in Haiti, a cholera outbreak began in Haiti’s Artibonite Department and rapidly spread across the country. As of March 18, 2012, a total of 531,683 cholera cases have been reported in Haiti, with 7056 deaths due to the epidemic (http://www.mspp.gouv.ht). Cholera cases were also reported in the Dominican Republic [1,2], and cases linked to the outbreak strain have been documented in travelers returning to their home countries from both Haiti and the Dominican Republic [1,3].

The absence of a previously recorded history of epidemic cholera in Haiti [4] raised interest in understanding the source of this outbreak. In order to further characterize the Haitian cholera strain, initial studies applied pulsed field gel electrophoresis and variable number tandem repeat typing to a large number of microbial isolates from the Haitian cholera outbreak [5,6]. These analyses identified the Haitian cholera strain as *V. cholerae* O1 El Tor, placing it as a seventh pandemic strain. In general, these studies found low levels of genetic variation in isolates, supporting a point-source origin for the outbreak [5-7].

More than a year has elapsed since *V. cholerae* was first introduced into Haiti. Identifying novel microbial variants that have emerged over the course of the outbreak may provide insight into the organism’s evolution on a short time scale. Genomic sequencing is the most powerful approach for evaluating such microbial evolution. Next-generation sequencing technologies, including Illumina, PacBio, and 454 sequencing, have increased the speed and decreased the cost of genome-wide sequencing. Chin et al. sequenced two *V. cholerae* isolates from Haiti using PacBio sequencing, which produces longer reads but has a higher error rate than other next-generation approaches [8]. Reimer et al. used single-end Illumina-based sequencing to sequence eight *V. cholerae* isolates from Haiti and one from the Dominican Republic [9]. Hendriksen et al. compared Haitian *V. cholerae* sequences to sequences from Nepal, finding that the Haitian isolates are highly similar to a set of isolates collected in Nepal in the summer of 2010 [10]. These sequencing studies indicated that the Haitian epidemic is most closely related to seventh pandemic strains from South Asia, and that the Dominican Republic outbreak strain is genetically nearly identical to the Haitian outbreak strain. The recent study of Hasan et al. [11] identified non-O1/O139 *V. cholerae* strains in patients in Haiti, and additional work is needed to explore the potential contribution of such strains to disease in Haiti.

In this study, we used paired-end Illumina sequencing at a high depth of coverage to sequence one *V. cholerae* isolate from the Dominican Republic, three isolates from Haiti, and three additional *V. cholerae* isolates. Four of the isolates were previously sequenced using a variety of sequencing technologies [8,12,13], and we present a comparison between sequence data generated using Sanger-based, next-generation, and PacBio sequencing technologies. The sequenced isolates include a classical O1-serogroup isolate from the sixth pandemic and an O139-serogroup strain as well as O1 El Tor strains from the seventh pandemic. The diverse strains sequenced and the high depth of coverage allow us to probe the sequence coverage required for optimal assembly and variant calling of the *V. cholerae* genome using next generation sequencing. Our data characterize the depth of coverage needed to accurately resolve sequence variation between *V. cholerae* strains.

We further identify sequence differences between the Haitian and Dominican Republic isolates in comparison to previously published and newly sequenced worldwide samples, and in comparison to each other. The three isolates from Haiti were collected in the same hospital in the Artibonite Department in October, 2010. The Dominican Republic isolate was collected three months later, in connection with a cholera outbreak among guests returning from a wedding in the Dominican Republic [1]. Since epidemic cholera had not been reported in Hispaniola prior to 2010, examining microbial mutations as the outbreak spread from Haiti to the Dominican Republic three months later provides insight into the temporal evolution of epidemic *V. cholerae*.

## Results and discussion

### Sequencing seven *V. cholerae* isolates at high depth of coverage

We sequenced seven *V. cholerae* isolates, including three isolates from Haiti (H1*, H2* and H3), one from the Dominican Republic (DR1), two from Bangladesh (N16961* and DB_2002), and one from India (O395*). Four of these isolates (H1*, H2*, N16961*, and O395*) were previously sequenced using a variety of sequencing technologies and to varying depths, and are denoted with an asterisk. We sequenced all strains to high depths of coverage (2643 – 5631x; Additional file 1: Table S1). We have deposited the sequence data in the Sequence Read Archive database (Submission: SRA056415).

### Effect of depth of coverage on genome assembly and single-nucleotide polymorphism (SNP) calling

**Figure 1:**
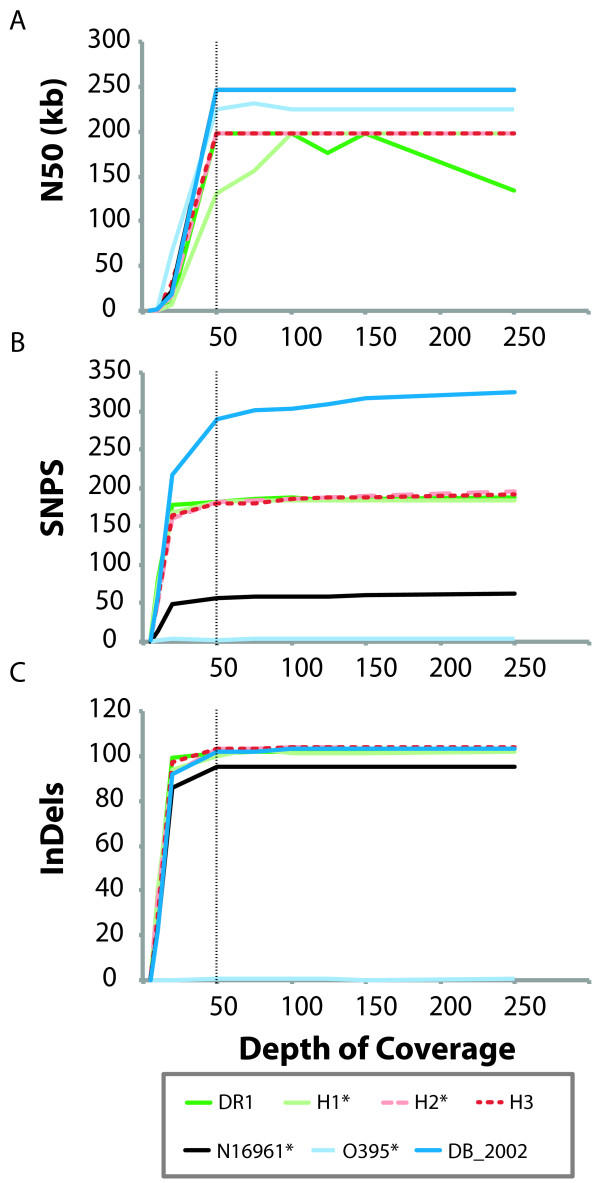
**Fiftyfold coverage suffices for whole-genome assembly and detection of most sequence varients.(A)** The N50 of the assembly, shown over a range of coverage depths (5x-250x), rapidly increases up to 50x coverage, and then plateaus. The median N50 of assemblies of five disjoint sets of reads at each depth of coverage is shown. **(B)** The number of SNPs detected increases rapidly up to 50x coverage, and gradually thereafter. **(C)** The number of insertions and deletions detected increases rapidly up to 20x coverage, and plateaus after 50x coverage. SNPs, insertions, and deletions in all isolates except for O395* are called relative to the N16961 genome [GenBank:AE003852, GenBank:AE003853]. For the O395* sample, due to the large number of differences (>20,000 SNPs) from the N16961 reference, SNPs, insertions, and deletions were identified instead against the Sanger-sequenced O395 reference [GenBank:CP000626, GenBank:CP000627].

The high depth of coverage of our sequencing enabled comparison of the efficacy of *de novo* assembly and variant detection at multiple depths of coverage. To assess the assembly quality, we used the N50 statistic. N50, a common metric of assembly quality, is the number of base pairs in the longest contig C such that fewer than half of the base pairs in the genome lie in contigs that are longer than C. We selected a random sample of the total reads for each isolate and compared the median N50 value for assemblies produced by Velvet at a range of coverage depths (5x to 250x), with three random read samples at each depth of coverage. For most isolates, N50 is stable across the range of depths from 50x to 250x, suggesting that 50x coverage is sufficient to construct a de novo assembly for these samples (Figure 1A). However, N50 continues to increase up to 100x coverage in sample H1*. The average read quality in H1* is the lowest of all the samples (Additional file 2: Figure S2), suggesting that while 50x is sufficient depth of coverage for de novo genome assembly on most samples, greater coverage is needed when average base quality is low.

We explored the effect of depth of coverage on calling sequence variants by examining the SNPs, insertions, and deletions identified at a range of coverage depths (5x to 250x). For all isolates, the number of SNPs identified increases sharply up to 50x coverage, and continues to increase gradually after this point (Figure 1B). In six of the seven isolates, at least 85% of the SNPs identified at 250x coverage are also identified at 50x coverage (the exception was the O395 sample, since at 50x coverage, we did not detect one of the three SNPs found at 250x coverage). SNPs identified uniquely at higher depths of coverage include variants in regions where the average base quality is low, regions with unusually low depths of coverage compared to the rest of the genome, and regions with false positive calls due to misalignment of reads across a deletion. Fifty-fold coverage is also sufficient to identify nearly all of the insertions and deletions observed at higher depths of coverage (Figure 1C). At 50x coverage, we detected at least 98% of the insertions and deletions observed at 250x coverage in each isolate. Twenty-fold coverage is sufficient to detect the majority of insertions and deletions; at least 90% of insertions and deletions that are observed at 250x coverage are also found at 20x coverage in five of the seven isolates. These results suggest that 50x coverage is sufficient to accurately call most variants, although deeper coverage provides additional power for identifying SNPs in some genomic regions.

### Comparison of sequence variants, insertions, and deletions identified using multiple sequencing approaches

Four of our isolates were previously sequenced using a variety of platforms. Those sequencing results provide an opportunity for us to compare variant calls across sequencing technologies, validate variant calls, and identify potential errors in reference sequences.

#### Comparison to N16961 Sanger reference sequences

**Figure 2:**
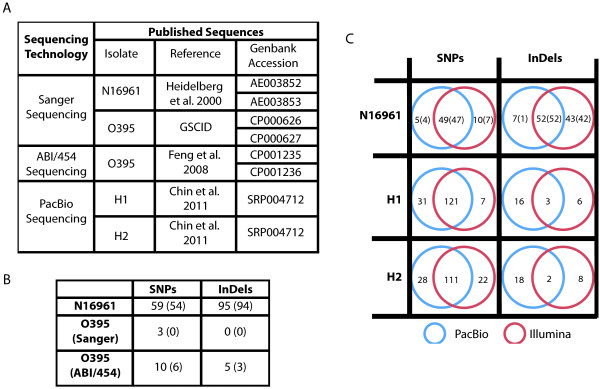
**Comparison of SNPs, insertions, and deletions called across sequencing technologies. (A)** List of published sequences for the four previously sequenced isolates (N16961, O395, H1, and H2) examined in this study. **(B)** Comparison of new Illumina sequences to GenBank references. The number of differences identified in the new sequence relative to the GenBank reference is shown in the table, with the number of differences confirmed by alignment to additional strains shown in parentheses. **(C)** Comparison of Illumina-based and PacBio-based SNP, insertion, and deletion calls relative to the Sanger-sequenced N16961 reference [GenBank:AE003852, GenBank:AE003853]. The number of variants called in PacBio sequencing only (red circle), in Illumina sequencing only (blue circle), or in both (intersection) are shown. For the N16961 sequences, the number of differences confirmed by alignment to additional strains is shown in parentheses. For H1 and H2, only variants that do not correspond to likely errors in the N16961 reference sequence are counted.

The original reference genome for *V. cholerae* was the Sanger-sequenced N16961 genome [12]. Feng et al. subsequently identified a number of corrections to the reference based on comparisons to additional strains at ambiguous positions and open reading frame clone sequence data [13]. Their corrections included 58 single base pair differences and 63 insertions and deletions. Similarly, we identified 59 single base pair differences as well as 95 insertions and deletions between N16961* and the N16961 reference [12] (Figure 2B).

To validate variant calls where the N16961* sequence differs from the corresponding reference, we examined the positions corresponding to those differences, using the Microbial Genome Browser alignment. Positions that differ between the reference sequence and the new isolates may represent errors in the reference sequence, false positive SNP calls, or mutations introduced during lab passage of the strains. If the discrepancy is due to an error in the reference sequence, then the sequences of additional strains in the alignment (O395 and MO10 for the N16961 sequence, N16961 and MO10 for the O395 sequence) are likely to agree with our variant call and disagree with the reference (Additional file 3: Figure S3). For 54 of the 59 differences, the alignments to strains O395 and MO10 support our new calls in N16961* (Additional file 3: Figure S3). Alignment to the additional strains supports all but one of the 95 insertions and deletions identified between N16961 and N16961*, consistent with the interpretation that the discordant positions correspond to errors in the reference sequence. We combined the corrections to the N16961 reference sequence previously identified by Feng et al. [13] with the validated variants that we identified to generate an updated list of sequence corrections (Additional file 4: Table S4).

#### Comparison to O395 Sanger and O395 ABI/454 sequences

To identify positions at which the sequence differed across multiple technologies, we compared the O395* sequence to the O395 Sanger and ABI/454-sequenced references ([GenBank:CP000626, GenBank:CP000627] and [GenBank:CP001235, GenBank:CP001236], respectively). We detected 3 SNPs between the O395* isolate and the Sanger-sequenced reference. BLAST queries indicated that in closely related strains, the sequence matches the reference at the position of these SNPs. However, manual examination of the SNP positions indicated that they are likely to be real variants, suggesting that they may have been introduced during laboratory passage of the O395 isolate (Additional file 5: Figure S5). We did not detect any insertions or deletions between the O395* sample and the O395 Sanger-sequenced reference. Between the O395* sequence and the ABI/454-sequenced O395 reference (Figure 2B), we detected seven additional single-base pair differences, four deletions, and one insertion. The accuracy of our Illumina calls at nine of these twelve positions is supported by their agreement with the Sanger-sequenced reference; for the other three positions, the Sanger-sequenced reference agrees with the ABI/454 calls.

#### Comparison to PacBio sequences

We compared three of the isolates that we sequenced (N16961*, H1*, and H2*) to previously published PacBio sequences for these same isolates (Figure 2C) [8]. In the N16961* sample, 83% of the SNPs that we identified (49/59 differences) were also present in the PacBio-based SNP calls. We identified ten SNPs not found in the PacBio variant calls, seven of which are validated by alignment to additional strains. Chin et al. reported five SNPs that we did not detect. Four of the five variants identified uniquely in the PacBio-based calls lie in repetitive regions of the genome, and these calls are supported by alignment to additional strains. The remaining SNP is not supported by alignment to additional strains. Although the majority of single nucleotide variant calls were consistent across platforms, only 55% of our Illumina-based insertions and deletions were also found using PacBio sequencing (52/95 indels). We identified 43 insertions and deletions in the N16961* sample not identified in the PacBio sequencing, and Chin et al. reported seven insertions and deletions that we did not recover. Only one of the seven insertions and deletions unique to the PacBio sequence is supported by alignment to additional strains, suggesting that the Illumina-based sequencing of the N16961 strain provided more sensitive and specific detection of insertions and deletions than the PacBio-based sequencing.

We also compared the variants identified in the H1 and H2 isolates relative to the N16961 reference by PacBio sequencing (H1, H2) with those identified by Illumina sequencing (H1*, H2*) (Figure 2C). Ninety-five percent (121/128) of the SNPs we identified in H1* were identified in the PacBio sequencing as well, while 83% (111/133) of the SNPs we called in H2* were also called in the PacBio sequencing. Thirty-one SNPs were identified uniquely in the PacBio sequencing of H1, while 28 SNPs were identified uniquely in the PacBio sequencing of H2. Many of the variant calls (11 in H1, 12 in H2) that were identified only by PacBio sequencing lie in repeat regions of the genome, suggesting that the long PacBio reads may facilitate detection of SNPs in repetitive regions of the genome that are difficult to recover using the shorter Illumina reads. Of the insertions and deletions that we identified in H1* and H2*, only 20-30% (3/9 for H1, 2/10 for H2) were also recovered in the PacBio-based calls. The PacBio-based sequencing identified 16 insertions and deletions in H1 and 18 in H2 not found in the Illumina-based calls. Thus, while both the Illumina-based and the PacBio-based sequencing identified similar SNPs, the insertion and deletion calls were highly divergent between the two approaches.

### Identifying SNPs, insertions, deletions, and structural variation across isolates

#### Analysis of an O139 serogroup isolate from Bangladesh

The O139 serogroup isolate from Bangladesh (DB_2002) was collected in Dhaka in 2002 and has not been previously sequenced. Relative to the N16961 reference strain, the isolate has deletions in the VPI-II genomic island, the superintegron, and a region on chromosome 1 associated with O antigen synthesis which contains genes involved in lipopolysaccharide and sugar synthesis/modification. The DB_2002 isolate contains two long regions that are absent from the N16961 reference. A 35,000-base pair region in the assembly of DB_2002 matches a region in an O139-serogroup strain from southern India that encodes genes for O-antigen synthesis [GenBank:AB012956.1]. The DB_2002 assembly also contains an 84,000-base pair region matching SXT integrative and conjugative element sequences in GenBank.

**Figure 3:**
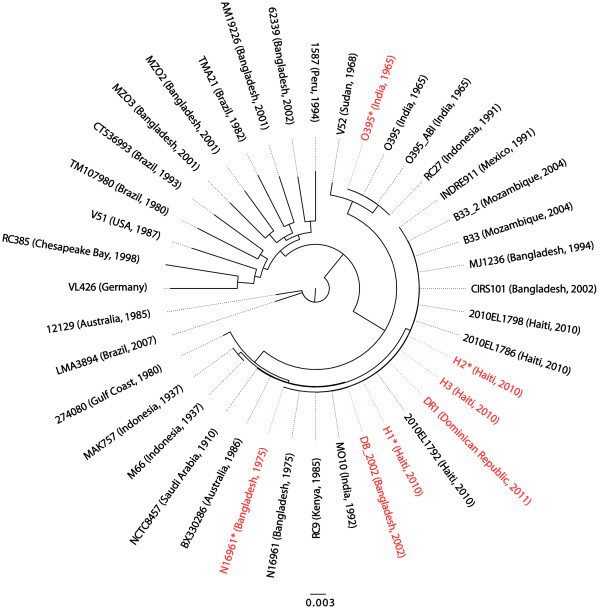
**Phylogeny of the sequenced strains and 33 previously sequenced *V. cholerae *isolates. ** We constructed a maximum-likelihood phylogeny using RaxML based on genes conserved across all newly sequenced isolates as well as 33 previously sequenced *V. cholerae * isolates. The isolates sequenced in our study are shown in red.

The genomic content of the DB_2002 isolate is similar to that of other O139 serogroup isolates. Phylogenetic analysis indicates that DB_2002 clusters closely with an O139 serogroup isolate from India (MO10, [GenBank: AAKF03000000]) (Figure 3). The deletions in the superintegron, absence of the VPI-2 genomic island, presence of the SXT region, and differences in O antigen genes are characteristic of other O139-serogroup isolates [14,15].

#### Analysis of Dominican Republic and Haitian isolates

The Haitian and Dominican Republic isolates cluster closely together and group in the phylogenetic tree with other seventh pandemic strains (Figure 3). Among the isolates in our phylogeny, the Haitian and Dominican Republic strains cluster most closely with strains from Bangladesh (CIRS101, [GenBank: ACVW00000000] and MJ-1236, [GenBank:CP001485, GenBank:CP001486]). In the alignments used to construct the phylogeny, there are an average of 12 substitutions between the newly sequenced Haitian/Dominican Republic isolates and CIRS101, and an average of 46 substitutions between the Haitian/Dominican Republic isolates and MJ-1236.

**Figure 4:**
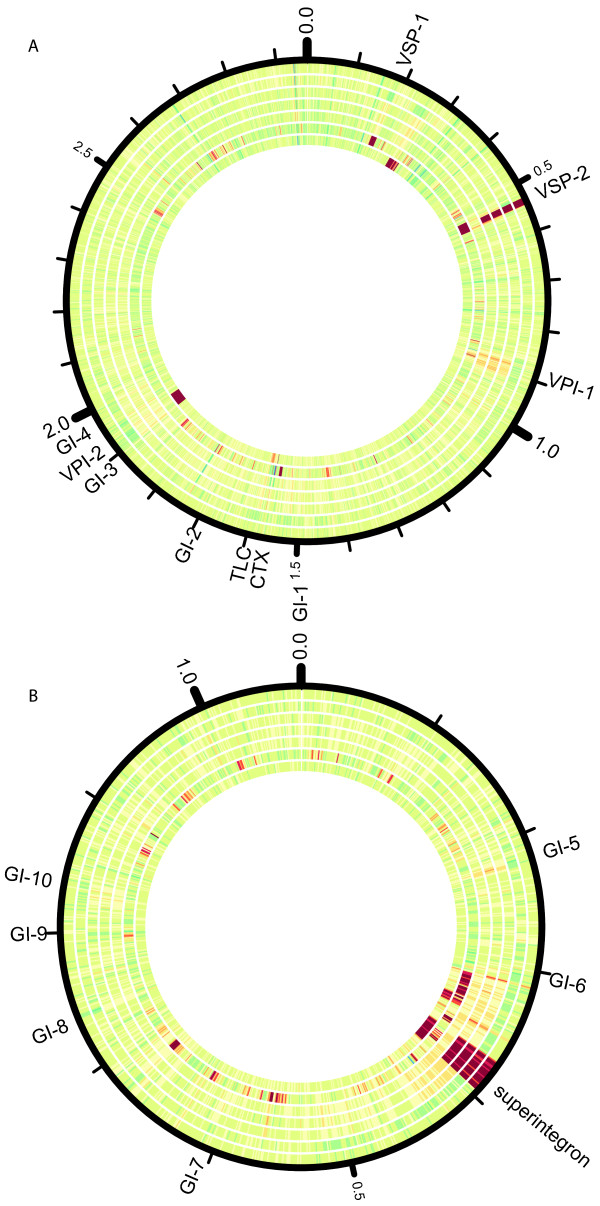
**Variation in depth of coverage of the sequenced isolates, based on read alignments of the seven sequenced strains against the N16961 reference genome.** Chromosome 1 **(A)** and chromosome 2 **(B)** are shown. The depth of coverage of 1000 base pair windows of 150x average coverage subsamples of the DR1 (outermost circle), H1*, H2*, H3, N16961*, O395*, and DB_2002 (innermost circle) isolates is displayed. Regions at low depth of coverage (<12x) are shown in red, while regions at high depth of coverage (>240x) are shown in blue. The depth of coverage in each window is displayed using the Circos tool [34]. Genomic islands as defined in [15] and the superintegron region as defined in [8] are shown.

To further characterize the Haitian and Dominican Republic isolates, we identified deletions and copy number variation relative to reference sequences (Figure 4). In all Haitian and Dominican Republic isolates, deletions were observed in the VSP-2 and superintegron regions. There are also deletions in the SXT region of the Haitian and Dominican Republic isolates relative to the MJ-1236 reference strain from Bangladesh (Additional file 6: Figure S6). To identify novel insertions, we aligned a 150x-coverage sample of N16961* reads to the *de novo* assembly of each Dominican Republic and Haitian isolate. All 1000-base pair windows in the *de novo* assemblies of the Haitian and Dominican Republic isolates to which N16961* reads did not map matched SXT integrating conjugative element sequences in GenBank, suggesting that no additional large insertions are present in the genomes of these isolates.

The four isolates from Haiti and the Dominican Republic are nearly identical in genomic sequence, consistent with a clonal origin for the epidemic. We identified three SNPs between the Haitian and Dominican Republic isolates, as well as one additional mutation in one of the Haitian isolates (Table 1). No sequence differences were identified between isolates H1* and H3, and no large-scale structural variation was observed across the Haitian and Dominican Republic isolates.

**Table 1:** Unique single nucleotide polymorphisms identified in individual Haitian and Dominican Republic cholera strains, in comparison to all other Haitian and Dominican Republic strains

**Isolate**	**Chromosome**	**Location**	**Ref Allele**	**Variant Allele**	**Associated gene**	**Type of change**
DR1	1	1565917/1572833*	T	C	*rstA*	Upstream of gene
H2*	2	166022	C	T	TagA-related protein	Nonsyn
DR1	2	467913	G	A	Pyruvate-flavodoxin oxidoreductase	Syn
DR1	1	3055641^†^	A	C	Transposase Tn3 family protein	Nonsyn

*The two locations provided for the *rstA*-related mutation correspond to the two copies of this gene in the N16961 reference strain.

†While all other genomic coordinates in the table are specified with respect to the N16961 reference strain, this variant lies in the SXT region, absent from the N16961 reference. Here, the genomic coordinates are specified with respect to the MJ-1236 reference.

#### Functional annotation of variants in Haitian and Dominican Republic cholera strains

The four isolates from Haiti and the Dominican Republic (DR1, H1*, H2*, and H3) are nearly identical in genomic sequence and share 126 variants relative to the N16961 reference. Seventy-three of these variants are non-synonymous mutations in coding genes (Additional file 7: Table S7). Notably, a number of the non-synonymous mutations occur in the same gene, or in genes with similar function, potentially indicating adaptive convergence. These include three mutations in the cholera enterotoxin (B subunit), and two mutations in MSHA biogenesis proteins (MshJ and MshE), which are involved in bacterial adhesion [16]. There are also two mutations that lie in two distinct DNA mismatch repair proteins, and two mutations in two outer membrane proteins, OmpV and OmpH.

In order to identify purifying or positive selection between the N16961 reference and the Haitian/Dominican Republic *V. cholerae* strains, we simulated random mutations in the cholera genome. To simulate random point mutations, we selected a genomic position uniformly at random, looked up the nucleotide at that position, and then randomly selected one of the three other possible bases at that position. We set the number of mutations equal to the number of differences between the N16961 reference and the Haitian/Dominican strains, and repeated the simulation 1000 times. At each iteration, we identified changes encoding non-synonymous substitutions (encoding a different amino acid than the original base, or a stop codon). When substitutions between each pair of nucleotides occurred with equal probability, synonymous changes were over-represented in the Haitian/Dominican Republic strains relative to the simulated data (p < 0.01), suggesting purifying selection. However, with transitions twice as likely as transversions, the enrichment of synonymous changes in the actual sequences relative to the simulation was not significant (p = 0.1).

We identified four mutations that occurred within the Haitian and Dominican Republic isolates (Table 1; Additional file 8: Figure S8), one in the SXT region, one in the CTX region, and two in the core genome. Three point mutations separated the Dominican Republic isolate from the Haitian isolates. These include a synonymous change in the pyruvate-flavodoxin oxidoreductase gene and a nonsynonymous substitution in transposase in the SXT region of the genome; both were also identified by Reimer et al. [9]. The third mutation separating the Dominican Republic and Haitian isolates is either within (according to [17]) or upstream (according to [GenBank:AE003852.1]) of the *rstA* gene, in the CTX region of the genome. The mutation upstream of *rstA* is in a region identified as bound by RstR in a DNAse I protection assay [17]. We also identified a non-synonymous mutation unique to one of the Haitian isolates in the *tagA*-related gene.

## Conclusions

The three Haitian isolates, the Dominican Republic isolate, and the other isolates that we have sequenced provide insight into the changes in *V. cholerae* over the course of the recent epidemic in Hispaniola. We identified four unique SNPs in individual Haitian and Dominican Republic cholera strains, in comparison to all other Haitian and Dominican Republic strains. One of these mutations is in the SXT region, one is in the CTX region, and two are in the core genome. These mutations include three mutations between the Haitian and Dominican Republic isolates, as well as one mutation unique to a single Haitian isolate. Our observation of three SNPs between isolates that are separated by three months is consistent with a recent estimate of an accumulation rate of 3.3 SNPs/year in the core *V. cholerae* genome [18].

The Haitian epidemic illustrates the transmission of *V. cholerae* across geographical boundaries. Multiple studies [8-10,19] have suggested that the Haitian cholera outbreak strain is likely to have originated in South Asia, and our analysis supports this conclusion. Clinical cases linked to the Haitian cholera strain have occurred in the Dominican Republic and in travelers who have recently visited the region. Thus, the use of whole-genome sequencing to trace the evolution of a strain involved in an ongoing outbreak is clinically relevant both for understanding an existing epidemic and for tracking related cases occurring in other regions.

Whole-genome sequencing of disease-causing organisms can reveal genetic differences between isolates that may be driven by adaption to new host or environmental factors. One of the mutations we identified between the Dominican Republic and Haitian isolates is in a region reported to be bound by the transcriptional repressor RstR [17], suggesting that this mutation might affect regulation of gene expression. This mutation is located upstream of the *rstA* gene, which is necessary for replication of the CTX phage genome [20]. The mutation in the Haitian isolate H2* is located in TagA-related protein. TagA-related protein is secreted extracellularly by *V. cholerae*[21] and is a homolog of TagA, which has mucinase function [22]. Sequencing of additional isolates from this outbreak over time is likely to provide further clues on the evolutionary dynamics of the *V. cholerae* genome.

Since even a single base pair mutation may have functional significance, accurate and complete detection of sequence variation is important. Understanding the effect of technical variables such as sequencing platform and depth of coverage is key to identifying genomic changes over the course of an epidemic. By sequencing to a high depth of coverage and re-sequencing strains that were previously sequenced using a variety of technologies, we were able to compare variant detection across multiple sequencing platforms and depths of coverage. We found that 50-fold coverage is sufficient for genome assembly and for the detection of most sequence variants, although some additional variants are detected at higher coverage depths. The majority of variant calls, insertions, and deletions are identified across the isolates regardless of sequencing technology. However, we also identified a set of sequence variants, insertions, and deletions that were observed uniquely in each platform. The high depth of coverage and low error rate of our Illumina sequencing permits accurate detection of sequence variants, insertions, and deletions. The long reads produced by the PacBio technology allows the identification of some additional variants, particularly in repeat regions. As increasing quantities of sequence data become available and new sequencing technologies emerge, further work will be needed to identify the effects of sequencing platform and analysis pipeline on the genome-wide identification of variants.

The increasing speed and decreasing cost of whole-genome sequencing permits the rapid characterization of microbial isolates over the course of an epidemic. Whole-genome sequencing can be used to track genomic evolution and functional variation in real time, to identify patterns of disease spread within a region, and to identify the source of an epidemic by tracing relationships to other strains around the world. Whole-genome sequencing is a powerful epidemiological tool whose applications towards understanding infectious disease are only beginning to be explored.

## Methods

### *V. cholerae* samples

We sequenced seven *V. cholerae* isolates. These samples include three clinical isolates from the cholera outbreak in Haiti isolated in October 2010, one clinical isolate from a cholera patient returning to the U.S. from the Dominican Republic isolated in January 2011, the *V. cholerae* O1 El Tor reference strain N16961 (Bangladesh, 1971 outbreak), the *V. cholerae* O1 classical reference strain O395 (India, 1965), and a 2002 *V. cholerae* O139 clinical isolate from Bangladesh (Table 2). The three Haitian isolates were all collected within days of each other in a single hospital in the Artibonite Department. Four of the seven samples have been previously sequenced using different sequencing technologies, and we denote these samples with an asterisk (*). Thus, we denote the samples from Haiti as H1*, H2*, and H3; the sample from the Dominican Republic as DR1; the samples from Bangladesh as N16961* and DB_2002; and the O1 classical reference strain from India as O395*.

**Table 2:** ***Vibrio cholerae *Isolates sequenced**

**Sample**	**Origin**	**Date**	***V. cholerae *serogroup and biotype**	**Previous sequencing method**
DR1	Dominican Republic	January 2011	O1 El Tor	
H1*	Artibonite Province, Haiti	October 2010	O1 El Tor	PacBio [8]
H2*	Artibonite Province, Haiti	October 2010	O1 El Tor	PacBio [8]
H3	Artibonite Province, Haiti	October 2010	O1 El Tor	
N16961*	Bangladesh	1971	O1 El Tor	Sanger [12], PacBio [8]
O395*	India	1965	O1 classical	Sanger (GSCID), ABI/454 [13]
DB_2002	Bangladesh	2002	O139	

An asterisk (*) denotes samples that have been previously sequenced.

### Sample preparation/isolation

We obtained clinical isolates (H1, H2, H3, DR1, DB_2002) from spontaneously passed human stool samples of patients with a diagnosis of cholera. All patients received standard medical treatment for cholera, appropriate to their medical condition. Bacteria were recovered from discarded stool specimens; no patient identifiers were collected and this was judged to be research exempt from human studies approvals by the appropriate Institutional Review Boards. Bacterial isolates were shipped from Haiti (H1, H2 and H3) and Bangladesh (DB_2002) to the U.S. following acquisition of appropriate licenses. DR1 is a clinical isolate from a cholera patient returning to the U.S. from the Dominican Republic. Isolates were confirmed as *V. cholerae* by standard biochemical assays and standard immunoagglutination assays. N16961 and O395 are common laboratory stock isolates (corresponding to ATCC 39315 and 39541 respectively) that have been maintained in glycerol at −80 degrees C.

### Illumina-based whole genome sequencing

We extracted DNA from *V. cholerae* strains using QiagenDNEasy (Qiagen, Valencia, CA). For Haitian strain H1* and Dominican Republic strain DR1, we fragmented samples by nebulization at 55 psi for four minutes. To isolate a 200 bp band, we ran the fragmented DNA on the Pippin Prep gel system (Sage Science, Beverly, MA). We processed samples H1* and DR1 using the commercial genomic DNA library preparation protocol (Illumina, San Diego, CA). Briefly, we end-repaired, 3’- adenylated, and adapter-ligated DNA fragments using standard Illumina adapters. We selected libraries by size and enriched by PCR for 15 cycles.

We received the remaining *V. cholerae* isolates (Table 2) at a later date and fragmented DNA from these isolates to approximately 200 bp using a Covaris shearing instrument. We prepared the fragmented DNA for sequencing using the commercial Illumina protocol for TruSeq DNA library preparations (Illumina, San Diego, CA). We selected libraries by size and enriched by PCR for 15 cycles to maintain consistency between methods.

We clustered the resulting libraries for all isolates in individual flow cell lanes and sequenced for 100 cycles on an Illumina HiSeq Analyzer, using paired-end technology. We filtered sequence reads based on quality scores. The resulting reads had high depth of coverage (> 2000x for each isolate when mapped to the N16961 reference genome using MAQ, a short read alignment tool [23]), enabling de novo assembly.

### De novo assembly

Using the Velvet genome assembler (v. 1.0.19) [24], we assembled the genomes on a subsample of reads from each isolate (69x-176x coverage when mapped using MAQ to the N16961 reference genome). We used the VelvetOptimiser script (version 2.1.17) to optimize the assembly parameters. We assessed the performance of the assembler on sets of reads at varying depths of coverage (Figure 1A).

### Comparison of sequence variants across sequencing technologies

We aligned subsamples of N16961* and O395* reads (150x coverage) to the corresponding published full genomes (Sanger-sequenced N16961 and Sanger-sequenced O395; Heidelberg et al., 2001, GSCID). We identified SNPs, insertions, and deletions as described above (Additional file 9: Table S9). We also compared the PacBio-based variant calls for isolates H1, H2, and N16961 [8] to variant calls for H1*, H2*, and N16961* (Figure 2A). To validate differences between the N16961* sequence and the N16961 published reference, we examined the alignment to additional strains using the Microbial Genome Browser [25]. Since the Microbial Genome Browser alignment track was not available for the O395 sequence, we used BLAST to examine the corresponding bases in related strains for positions at which the O395* sequence differed from the Sanger-sequenced O395 reference.

### Identifying SNPs, insertions, deletions, and structural variation across isolates

We called SNPs, insertions, and deletions on three non-overlapping 150x subsamples of reads. SNPs, insertions, and deletions shared among all three subsamples are reported here (Additional file 10: Table S10). Using the BWA short-read aligner [26], we aligned each 150x read subsample to the N16961 reference genome [GenBank:AE003852, GenBank:AE003853]. For the O395* sample, we aligned instead against the Sanger-sequenced O395 reference [GenBank:CP000626, GenBank:CP000627].

We recalibrated base quality scores and performed realignment around insertions and deletions using the Genome Analysis Toolkit, a framework for analyzing next-generation sequence data [27]. We called SNPs using the variant detection tool Varscan [28], requiring a minimum SNP frequency of 25% to allow for SNP calling in repeat regions of the genome. To reduce sequencing artifacts, we required that the variant call be represented on reads in both directions, with no more than three-quarters of the variant calls on reads in the same direction when fewer than 90% of the reads carried the variant call.

We identified small insertions and deletions on the realigned, recalibrated pileup files (aligned to the N16961 reference genome) using Varscan, requiring a 75% variant frequency. To restrict the variant set to differences with the reference genome, we removed variants identified between the N16961* isolate and the N16961 reference. For functional annotation of SNPs, we used the snpEff software [29].

To identify large-scale structural variants, we examined variation in the depth of coverage in 1000-base pair windows when a sub-sample of the reads was aligned against the N16961 and MJ-1236 [30] reference genomes, similar to the approach in Chin et al. [8]. To identify large insertions relative to the N16961* genome, we used MAQ to align a 150x-coverage subsample of the N16961* reads to the *de novo* assembly for each isolate. We characterized all thousand base pair windows without aligned reads using a BLASTn search against the “nr/nt” database.

In order to identify high-confidence sequence differences across the Haitian and Dominican Republic isolates, we used Fisher’s exact test based on counts of reads aligned at each position to the N16961 and MJ-1236 reference genomes, similar to the approach implemented in the Nesoni tool [31]. We eliminated reads with quality scores with a greater than 1% estimated error rate from the count, as well as positions at which more than three-quarters of variant calls were on reads in the same direction. We removed variant calls based on sequence reads with multiple differences from the reference as well as at positions where more than a quarter of the reads in both isolates carried the variant call. We reported high-confidence SNPs with Bonferroni-corrected *p* < 0.01.

### Constructing a phylogeny

To construct a phylogeny, we identified genes conserved across all newly sequenced isolates as well as 33 previously sequenced *V. cholerae* isolates (Additional file 11: Table S11). We included all genes for which the top BLAST hit to the N16961 reference gene had at least 70% identity in all strains. To eliminate paralogs, we required the next best hit to be less than 0.8 times as similar as the best hit. We constructed a multiple sequence alignment for the nucleotide sequences of the 1740 genes meeting these criteria using the multiple sequence alignment tool MUSCLE [32]. We concatenated the alignments of genes present in all strains, and constructed a maximum-likelihood phylogeny with RaxML [33], using the General Time Reversible model of nucleotide substitution.

## Competing interests

The authors declare that they have no competing interests.

## Authors’ contributions

PCS, JH, and RCL conceived of the study. RS performed the computational analysis and drafted the manuscript. PCS, SG, RCL, and JH helped to draft the manuscript. SG prepared the paired-end libraries for sequencing and CE performed experiments. PCS and MK supervised the study. SC, FQ, LH, ETR, RCL, and JH obtained microbiologic samples. All authors read and approved the final manuscript.

## Supplementary Material

Additional file 1 **Table S1.** Depth of coverage and number of reads for each sequencing lane. Click here for file

Additional file 2 **Figure S2.** Quality score vs. sequencing cycle for each isolate. Click here for file

Additional file 3 **Figure S3.** Example alignments to additional strains for the validation of SNPs and insertions/deletions identified in N16961* relative to the N16961 reference. Click here for file

Additional file 4 **Table S4.** Corrections to the N16961 reference sequence. Click here for file

Additional file 5 **Figure S5.** Read alignment at positions at which the O395* isolate differs from the corresponding reference sequence. Click here for file

Additional file 6 **Figure S6.** Alignment of the seven sequenced isolates against the MJ-1236 reference genome. Click here for file

Additional file 7 **Table S7.** Non-synonymous SNPs shared among Haitian and Dominican Republic isolates. Click here for file

Additional file 8 **Figure S8.** Alignments of DR1 reads at the positions where DR1 and Haitian cholera isolates differ. Click here for file

Additional file 9 **Table S9.** Counts of SNPs, insertions, and deletions identified in each isolate relative to the N16961 reference. Click here for file

Additional file 10 **Table S10.** SNPs and indels in sequenced isolates. Click here for file

Additional file 11 **Table S11.** List of strains included in phylogenetic analysis. Click here for file
