# Supplementary material for: High depth, whole-genome sequencing of cholera isolates from Haiti and the Dominican Republic
Source: BMC Genomics. 2012 Sep 11;13:468. doi: 10.1186/1471-2164-13-468 (PMC3473251; doi:10.1186/1471-2164-13-468)

Supplementary Figure S2: Quality score vs. cycle. The average reported quality score of a 150x subsample of reads for each isolate is shown.

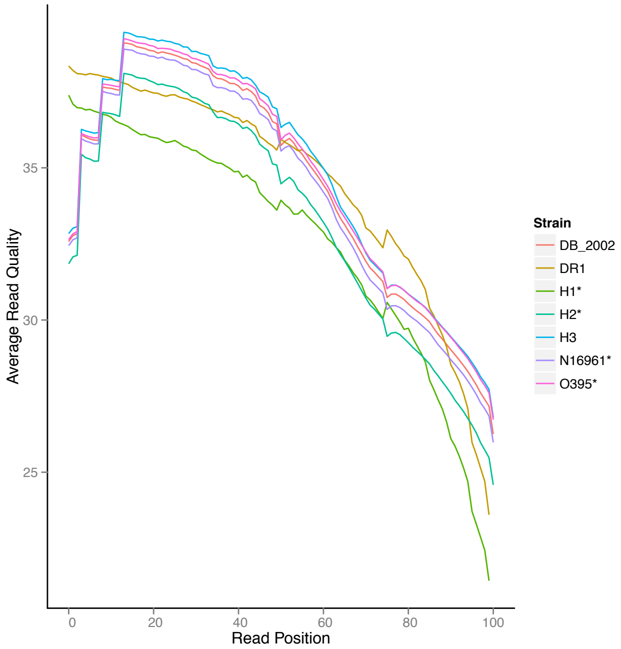

Supplement: Additional file 2 — Figure S2. Quality score vs. sequencing cycle for each isolate. [file 1471-2164-13-468-S2.pdf]
