# Supplementary material for: High depth, whole-genome sequencing of cholera isolates from Haiti and the Dominican Republic
Source: BMC Genomics. 2012 Sep 11;13:468. doi: 10.1186/1471-2164-13-468 (PMC3473251; doi:10.1186/1471-2164-13-468)

Supplementary Figure S3: Example alignments to additional strains for the validation of SNPs and insertions/deletions identified in N16961* relative to the N16961 reference. In order to validate SNP calls in the N16961* isolate that differ from the Genbank reference, we examined the alignment in the Microbial Genome Browser of the N16961 reference to additional strains. If the most closely related additional strains match the variant call and differ from the N16961 Genbank sequence, the alignment supports our variant call. The aligned bases from top to bottom are from N16961, O395, M010, V. vulnificus YJ016, V. vulnificus CMCP6, V. parahaemolyticus, and V. fischeri ES114. (A) shows a genomic position, chr1:424,629-424,629, at which we identify a SNP in the N16961* isolate, and the alignment to additional strains supports our base call of "T." (B) shows a genomic position, chrI:359,984-359,986, where we identify a one base pair long insertion which is supported by alignment to additional strains. The images are screenshots from the Microbial Genome Browser.

A. chrI:424,629-424,629

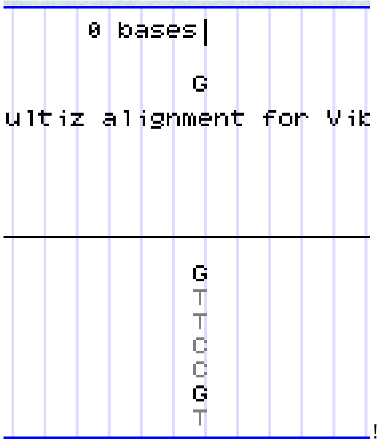

B. chrI:359,984-359,986

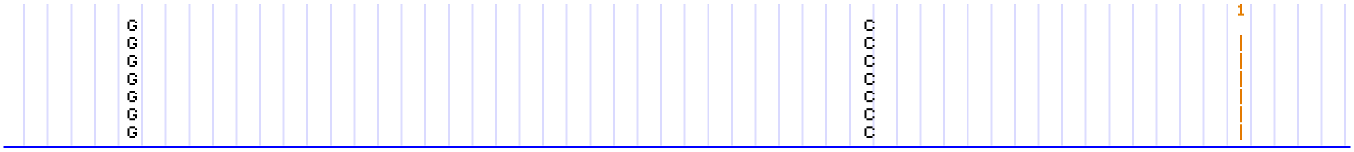

Supplement: Additional file 3 — Figure S3. Example alignments to additional strains for the validation of SNPs and insertions/deletions identified in N16961* relative to the N16961 reference. [file 1471-2164-13-468-S3.pdf]
