# Supplementary material for: High depth, whole-genome sequencing of cholera isolates from Haiti and the Dominican Republic
Source: BMC Genomics. 2012 Sep 11;13:468. doi: 10.1186/1471-2164-13-468 (PMC3473251; doi:10.1186/1471-2164-13-468)
Supplement: Additional file 5 — Figure S5. Read alignment at positions at which the O395* isolate differs from the corresponding reference sequence. [file 1471-2164-13-468-S5.pdf]

A.

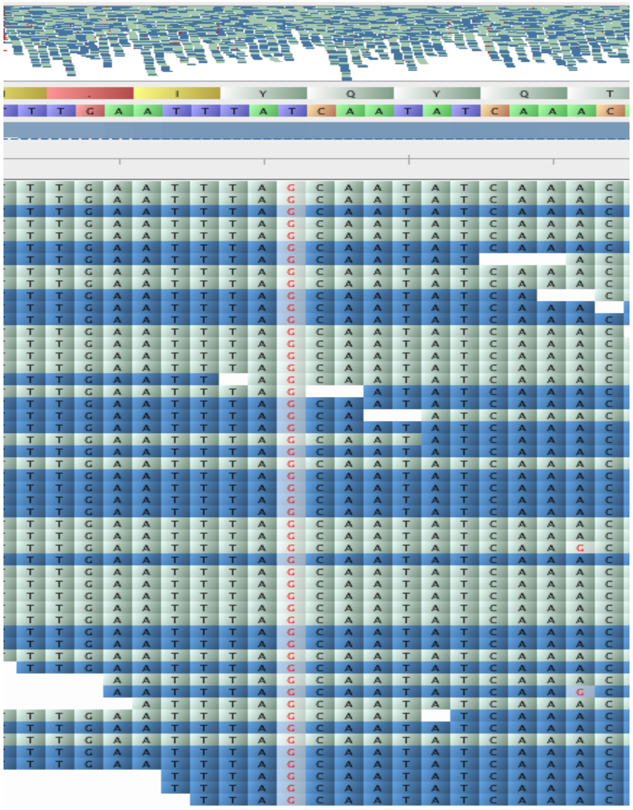

C.

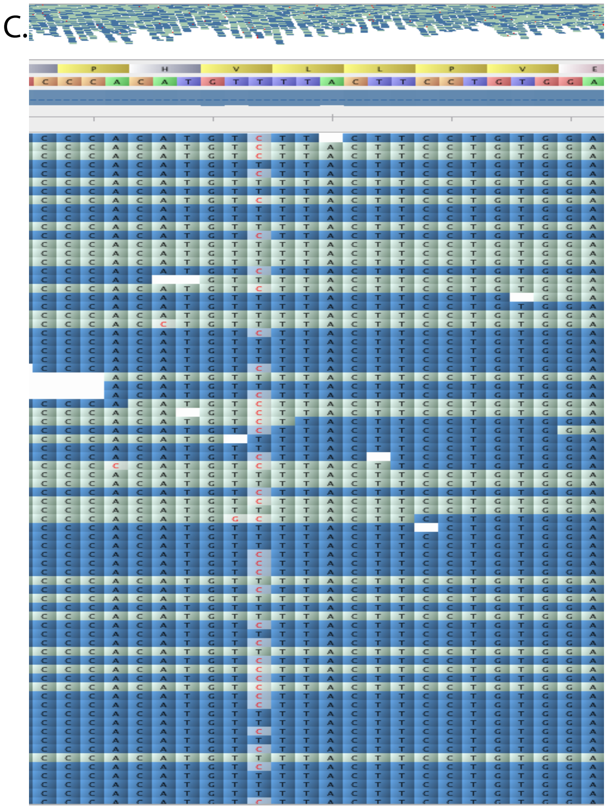

B.

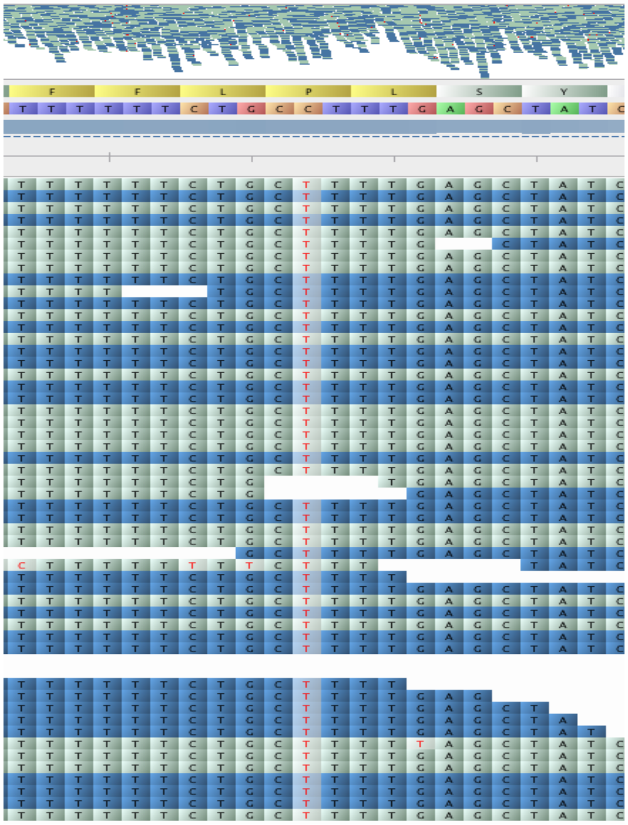

Supplementary Figure S5: Read alignment at positions at which O395* isolate differs from the Genbank reference sequence. A. The SNP is on chromosome [Genbank:CP000627.1], at position 449946, and consists of a T to G point mutation. B. The SNP is on chromosome [Genbank:CP000627.1], at position 540932, and consists of a C to T point mutation. C. The SNP is on chromosome [Genbank:CP000627.1], at position 2485347, and consists of a T to C point mutation in a subset of the reads. The location is an intragenic spacer region between two tRNA genes. Although only a subset of reads carry the SNP, the reads with the SNP are equally distributed in both read directions, and read pairs that both overlap the base either both carry the SNP or both carry the reference base, suggesting that the SNP is likely to correspond to a real sequence difference. Read alignments were viewed using the Tablet program (Milne et al., Tablet—next generation sequence assembly visualization, Bioinformatics, 2009).
