# Supplementary material for: High depth, whole-genome sequencing of cholera isolates from Haiti and the Dominican Republic
Source: BMC Genomics. 2012 Sep 11;13:468. doi: 10.1186/1471-2164-13-468 (PMC3473251; doi:10.1186/1471-2164-13-468)

Supplementary Figure S6: Alignment of seven sequenced isolates against the MJ-1236 reference genome (chromosome 1, left; chromosome 2, right). Inward from the outer circle, the depths of coverage of 1000 base pair windows of a 150x average coverage subsample of the DR1, H1*, H2*, H3, N16961*, O395*, and DB_2002 isolates are shown. Regions at low depth of coverage (< 12x) are shown in red, while regions at high depth of coverage (>240x) are shown in blue.

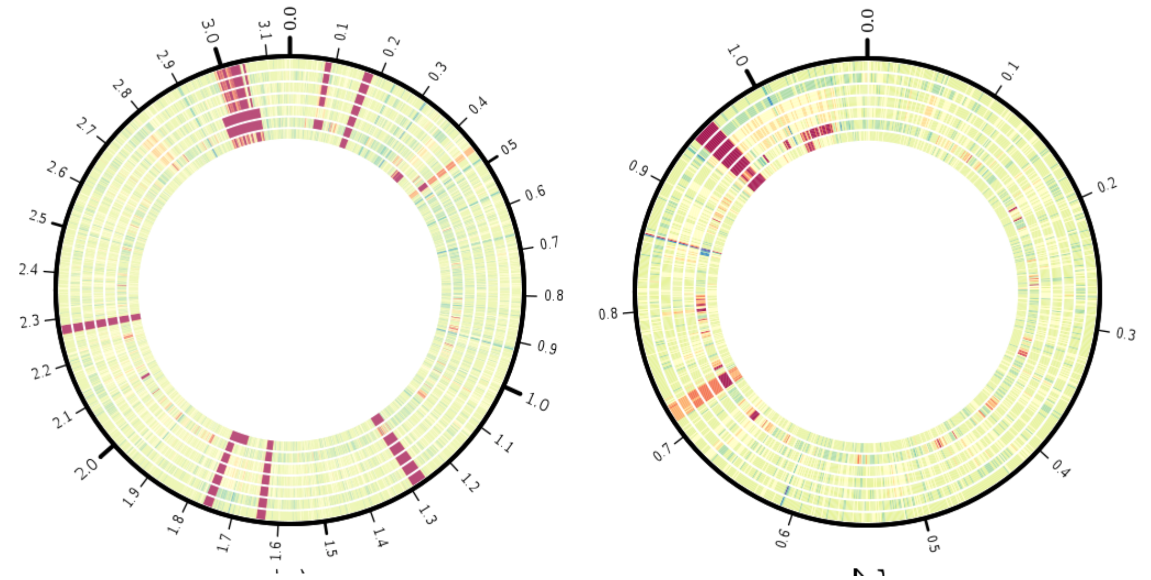

Supplement: Additional file 6 — Figure S6. Alignment of the seven sequenced isolates against the MJ-1236 reference genome. [file 1471-2164-13-468-S6.pdf]
