# Supplementary material for: High depth, whole-genome sequencing of cholera isolates from Haiti and the Dominican Republic
Source: BMC Genomics. 2012 Sep 11;13:468. doi: 10.1186/1471-2164-13-468 (PMC3473251; doi:10.1186/1471-2164-13-468)
Supplement: Additional file 8 — Figure S8. Alignments of DR1 reads at the positions where DR1 and Haitian cholera isolates differ. [file 1471-2164-13-468-S8.pdf]

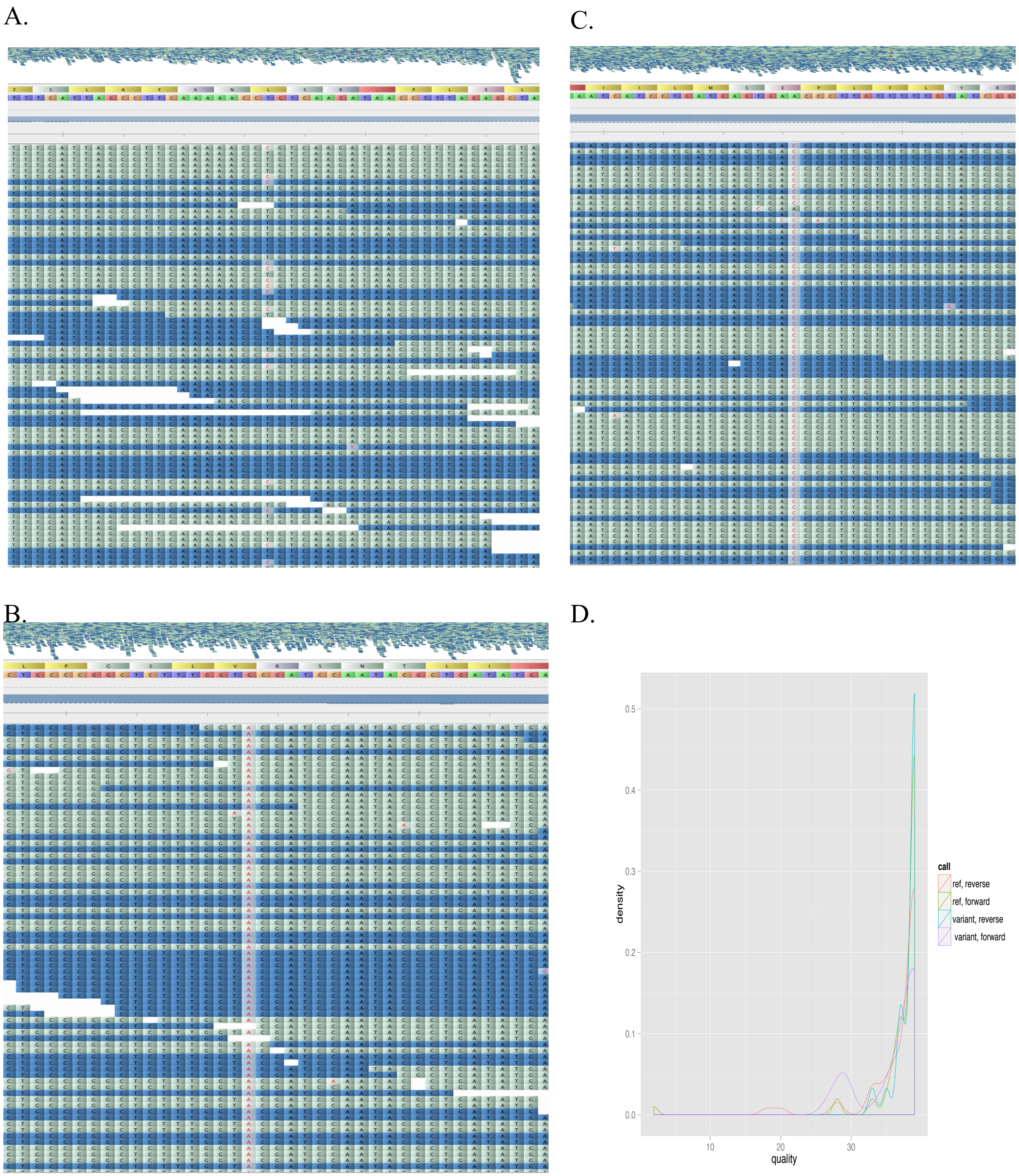

Supplementary Figure S8: Alignments of DR1 reads at the positions where DR1 and Haitian cholera isolates differ. Alignments for A and B are relative to the N16961 reference strain; alignment C is relative to the MJ1236 reference strain. (A) Alignment around SNP at chr1, base 1565917. Although only a subset of reads carry the SNP, the reads with the SNP are equally distributed in both read directions. For paired reads where both reads in the pair overlap the base, either both carry the SNP or both carry the reference base, suggesting that the SNP is likely to correspond to a real sequence difference. Additionally, the SNP occurs near the RSTA gene, which is present in two copies in the N16961 genome, once in the CTX locus and once in the RS1 locus, suggesting that the mutation may occur in one copy of the gene in the Dominican Republic *V. cholerae* genome. Alternate explanations for the mixture of base calls in this position include heterogeneity in the sequenced bacteria at this locus and sequence error. Variant reads at this position were observed in both replicate lanes for the DR1 isolate, and were not observed in the alignment of reads from the Haitian isolates to the N16961 reference. (B) Alignment around SNP at chr2, base 467913. (C) Alignment around SNP at chr1, base 3055641 (MJ-1236 reference). Read alignments were viewed using the Tablet program (Milne et al., Tablet—next generation sequence assembly visualization, Bioinformatics, 2009). D. At the reads aligning to chr1:1565917, quality scores are similar for reads carrying the reference base as for reads carrying the variant call. The quality scores shown are for a random 150x subsample of reads.
